# A career in science requires a thick skin

**DOI:** 10.1038/s44319-025-00477-7

**Published:** 2025-05-20

**Authors:** David R Smith

**Affiliations:** Department of Biology, Biological & Geological Sciences Bldg., Western University, 1151 Richmond Street, London, N6A 5B7 ON Canada

**Keywords:** Careers

## Abstract

Academic science is a bumpy road with many setbacks and students should be better prepared for losses and failures along the way.

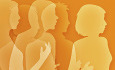

*“I have a thick skin, but I have a heart.” – Dan Savage*

I spent the winters of my youth traveling across Canada competing in cross-country ski races. Being the shortest kid in my high school, I was not designed at all to propel myself across snowy fields with a pair of long poles. But I donned my Lycra and persevered. As I watched my teammates win national—and eventually world—championship medals, I conceded that no matter how hard I trained, I would remain at the back of the pack, a fluorescent Nordic caboose. This was a lesson in humility, propulsion physics and human genetics, and one that I hold my stalky, five-foot-four father wholly responsible.

Although I did not appreciate it at the time, my experiences on the ski trails would serve me well on the journey to become a scientist. Navigating graduate school, postdoctoral studies, the academic job market and the peer-review process is no easy feat and can often feel like a deluge of disappointments rather than a series of successes. Again, I blame my father for this, for being so darn kind and encouraging to me throughout my adolescence. He never once sat me down and said: “Son, I hate to tell you this, but you might not be as gifted as your mom and I have led you to believe.”

Kidding aside, one would be forgiven for thinking that the scientific endeavour is one of continual accomplishments. Click on the homepage of any major academic institute, and what do you see? A bragging board of achievements. “Multimillion dollar grant!” “Another major breakthrough!” “Prestigious award!” “Record-breaking citations!” I guess I should be relieved that the headlines do not read: “Professor Smith strikes out again!” or “Celebrating a series of low-impact papers”. But at least these kinds of headlines would give students a sense of what it is really like on the frontlines of research.

If I could distill my scientific life into five words, they would read: “I regret to inform you”. I know this phrase so well that I can spot it even when it’s veiled beneath more uplifting language: “Dear David. It’s my pleasure to tell you that you’ll be teaching the two-thousand-person second-year genetics course next term.” These days, when I open emails, my eyes go directly to any evil looking buts, like “What an amazing pool of applicants! Yours was among the very best, but…” The worst, however, is no words at all. “Hey Dave, did you ever hear back about that society award?” “Well, it’s been two years, but I still have hope.”

Shortly after I completed my PhD, a faculty position in my field opened at a small liberal arts college where I’d taken my undergrad. I dreamt of returning to this quaint little campus, to the department where I’d fallen in love with genetics and bonded with many of the faculty. I immediately submitted my application and then started checking my email incessantly for a response. The months tiptoed by; I never received a reply. Years later, when I gave an invited talk at that same university, I was kind and courteous but crying on the inside. If any departmental chairs are reading this, please take the time to respond to job applicants. Even a form letter with those five infamous words is better than cold, dark silence.

Sometimes you think you have triumphed only to discover that it’s really a blunder. One of the first essays I wrote for *EMBO reports*—which, back then, was a subscription journal—following the 2016 US election, was titled: “More than ever, scientists need to engage with the public: the stakes are high and they may be for keeps.” Shortly after the article went online, I noticed it was garnering a lot of attention on Twitter. I shouted to my wife: “One of my articles has gone viral!” “For good or bad reasons?” she asked. Bad reasons, I mumbled to myself, of course not. Then I had a closer look at the comments. An influential science social media influencer had tweeted: “Unintentionally ridiculous: calling for #scientists to engage with the public in an article that is behind an expensive paywall!!!” The other comments were all equally as harsh. The pushback got so bad that the journal eventually made the essay open access. To this day, it remains among the most popular articles I’ve written. If only it were for the right reasons.

Reading reviewers’ reports can have a particularly painful sting. There’s a folder on my laptop computer called *Thanks for coming out* where I store my favourites. There’s nothing like pouring three years of brainpower into a research paper only to be told that “you would be best served by refocusing your efforts on something more constructive.” I guess that’s better than: “Certainly among the worst papers I’ve had the misfortune to read.” I think my wife would side with reviewer number 3 on this one: “It is impressive that the author can take something so intuitively interesting and make it so incredibly boring.” “Has the author considered submitting to a smaller, more specialized journal?” Yes, I have.

I write all of this because I believe scientists should be more forthcoming about their losses and failures. We should let students skim through our *Thanks for coming out* folders, learn about the interviews we flunked, the manuscripts that lay in waste, and the grants that were burned at the stake. Students often see the careers of their professors and mentors as the direct outcome of a long chain of uninterrupted accomplishments rather than the circuitous, setback-ridden road that most have endured.

It does not help that there is a growing trend to shield young people from failure. In Canada, for example, at youth soccer tournaments there is a trend to give everyone a medal, even the losers. And it is well documented that the Canadian education system is inching towards a no-fail policy. If we are to shield our children and students from failure and rejection, then we should at least make them aware of the cutthroat work environments that ultimately await them—environments where some, and more likely a lot, of failure is guaranteed.

I have an old photo album detailing my early years as a cross-country skier. On the inside cover is a faded sticky note that my old ski coach, Dave Battison, stuck on my high-school locker after a particularly brutal performance. It says, “Buck up, Smitty. You may be short and slow, but when they knock you down, get up and go.” Good advice for anyone navigating the blistery trails of work and life.

## Supplementary information

Peer Review File

