# Supplementary material for: A career in science requires a thick skin
Source: EMBO Rep. 2025 May 20;26(12):2971–2. doi: 10.1038/s44319-025-00477-7 (PMC12187916; doi:10.1038/s44319-025-00477-7)
Supplement: Supplementary file 1 — Peer Review File [file 44319_2025_477_MOESM1_ESM.pdf]

EMBO reports

A career in science requires a thick skin

David Smith

Corresponding author(s): David Smith (dsmit242@uwo.ca)

**Review Timeline:**	Submission Date:	8th May 25
	Accepted:	9th May 25

Editor: Holger Breithaupt

Transaction Report: This article type is not peer reviewed.
